# Long-Term Effects of White-Tailed Deer Exclusion on the Invasion of Exotic Plants: A Case Study in a Mid-Atlantic Temperate Forest

**DOI:** 10.1371/journal.pone.0151825

**Published:** 2016-03-28

**Authors:** Xiaoli Shen, Norman A. Bourg, William J. McShea, Benjamin L. Turner

**Affiliations:** 1 Conservation Ecology Center, Smithsonian Conservation Biology Institute, National Zoological Park, Front Royal, VA, United States of America; 2 Smithsonian Tropical Research Institute, Balboa, Ancon, Republic of Panama; Fudan University, CHINA

## Abstract

Exotic plant invasions and chronic high levels of herbivory are two of the major biotic stressors impacting temperate forest ecosystems in eastern North America, and the two problems are often linked. We used a 4-ha deer exclosure maintained since 1991 to examine the influence of a generalist herbivore, white-tailed deer (*Odocoileus virginianus*), on the abundance of four exotic invasive (*Rosa multiflora*, *Berberis thunbergii*, *Rubus phoenicolasius* and *Microstegium vimineum*) and one native (*Cynoglossum virginianum*) plant species, within a 25.6-ha mature temperate forest dynamics plot in Virginia, USA. We identified significant predictors of the abundance of each focal species using generalized linear models incorporating 10 environmental and landscape variables. After controlling for those predictors, we applied our models to a 4-ha deer exclusion site and a 4-ha reference site, both embedded within the larger plot, to test the role of deer on the abundance of the focal species. Slope, edge effects and soil pH were the most frequent predictors of the abundance of the focal species on the larger plot. The abundance of *C*. *virginianum*, known to be deer-dispersed, was significantly lower in the exclosure. Similar patterns were detected for *B*. *thunbergii*, *R*. *phoenicolasius* and *M*. *vimineum*, whereas *R*. *multiflora* was more abundant within the exclosure. Our results indicate that chronic high deer density facilitates increased abundances of several exotic invasive plant species, with the notable exception of *R*. *multiflora*. We infer that the invasion of many exotic plant species that are browse-tolerant to white-tailed deer could be limited by reducing deer populations.

## Introduction

Long-term study of the temperate forest ecosystem in the Mid-Atlantic region of eastern North America has revealed a large decline of biodiversity and decrease in abundance of native plant species [1–3]. Among the several driving mechanisms, chronic high herbivore abundance and exotic plant invasion are two stressors causing the increase of homogeneity of local plant communities, especially the forest understory species component [2,4]. The density of white-tailed deer (*Odocoileus virginiaus* Zimmermann, henceforth deer) has dramatically increased throughout the region since European settlement, and has chronically exceeded historical levels in many forests [5]. Deer consume a large amount of plant biomass (i.e., leaves, buds, flowers and fruits) and directly reduce the growth, reproduction and survival of herbaceous and woody plant species. By foraging selectively, deer can change the relative abundance and thus alter competitive relationships of plant species [6]. Chronic high deer density reduces understory plant cover and diversity, impedes the regeneration of seedlings and saplings, alters nutrient and carbon cycling, and eventually may redirect succession of canopy tree species [7,8]. Sustained heavy browsing also exerts cascading effects on invertebrates, birds, and other mammals by altering resource availability at other trophic levels [9]. Many studies have demonstrated that deer may act as a keystone species, strongly affecting the structure and functioning of the temperate forests of eastern North America [5].

Mid-Atlantic temperate forests have suffered from the invasion of exotic plant species for over 100 years [10]. Several herbaceous and woody exotic species have established in many of these forests, such as garlic mustard (*Alliaria petiolata*), Japanese stiltgrass (*Microstegium vimineum*), multiflora rose (*Rosa multiflora*), wineberry (*Rubus phoenicolasius*), Japanese honeysuckle (*Lonicera japonica*) and Japanese barberry (*Berberis thunbergii*). These exotic species were naturalized in the early 20^th^ century and expanded out from areas adjacent to human development into intact forest. They compete for resources with native plants and indirectly suppress the growth of native plants by changing soil chemistry, microbial community dynamics, nutrient cycling and biotic interactions [11,12], resulting in profound effects on the composition and function of native plant communities.

Recent experimental studies show that these dual stressors, deer and exotic invasive plants, may be linked, as invasive plants decrease in abundance when deer are excluded from forest communities or maintained at low densities [13–15]. Previous studies have found deer may facilitate the dispersal and colonization of exotic invasive plants by epizoochory (seeds transported externally by animals) and endozoochory (seed dispersal via ingestion) [16,17]. They may also facilitate invasion, through either competitive release following selective consumption of native species [14,18] or soil and litter disturbance resulting from foraging and bedding [19]. Exotic plants that are unpalatable and/or browse-tolerant, a common characteristic of many invasive plants, may have a competitive advantage over browsed native plants [20]. Facilitation of exotic plant invasion by extensive deer browsing has been observed by both short-term (e.g., 3-year deer exclusion by Eschtruth and Battles 2009 [13] and 6-year deer exclusion by Kalisz et al. 2014 [14]) and long-term experimental studies (e.g., 13-year deer exclusion by Kuebbing et al. 2013 [21]; 18-year deer exclusion by Abrams and Johnson 2012 [22]).

Here, we report a case of a long-term deer exclusion experiment to examine the impact of white-tailed deer browsing on the abundance of four exotic invasive plant species (three woody: *R*. *multiflora*, *B*. *thunbergii*, *R*. *phoenicolasius*; one herbaceous: *M*. *vimineum*), all widely found throughout the Mid-Atlantic region, at a large (25.6 ha) forest plot located in Virginia, USA. We investigated the abundance of these exotic invasive species and the environmental characteristics under which they occurred. After controlling for potential confounding factors, we compared the abundance of these species in a 4-ha fenced deer exclusion area established in 1991, and a paired 4-ha reference site, both found within the larger plot. Our study has value in being long-term due to the slow process by which deer impact native plant communities [3]. In addition, exotic species invasion processes progress at slower rates in closed-canopy forests where the light levels are consistently low and the lag-time to invade the forests is generally long [23,24], so short-term resistance to invasive species may not accurately assess their impact. We also used a common native herbaceous species, wild comfrey (*Cynoglossum virginianum*), that is known to be deer-dispersed and unpalatable to deer [25], as a comparison species. While the lack of replication limited the scope of inference of our study, our exclosure and reference sites are larger than most exclosures, and we could take advantage of highly detailed environmental data from a large forest plot and a long-term deer exclusion experiment.

## Materials and Methods

### Study site

This study was conducted at the 25.6-ha SCBI large forest dynamics plot (38°53’ N, 78°9’W) located at the Smithsonian Conservation Biology Institute (SCBI), approximately 5 km south of Front Royal, VA, USA (see Bourg et al. 2013 [26] for details). Mean elevation of the SCBI plot is 302 m (range 273–338 m). The 2011 mean annual temperature was 12.7 ± 0.66°C and the mean annual precipitation was 96.2 ± 15.8 cm based on climate data obtained from a nearby weather station (Carr, University of Virginia, 2011 unpublished data). The dominant soil series in the plot are Myserville and Montalto, which are stony, steep, and well-drained. The vegetation of the SCBI plot represents a typical mature secondary mixed deciduous forest in the mid-Atlantic region of eastern North America, with overstory tree ages ranging from 84 to 124 years [8]. Landform variation across the plot supports upland and bottomland forests. Its woody vegetation was censused in 2008, using standardized methods [27], with all free-standing stems ≥ 1 cm DBH (Diameter at Breast Height) identified, measured, tagged and mapped. Dominant canopy trees include tulip poplar (*Liriodendron tulipifera*), white, red and black oak (*Quercus alba*, *Q*. *rubra*, and *Q*. *velutina*), white ash (*Fraxinus americana*), pignut and mockernut hickory (*Carya glabra* and *C*. *tomentosa*), and black gum (*Nyssa sylvatica*). Prominent understory components include spicebush (*Lindera benzoin*), paw-paw (*Asimina triloba*), American hornbeam (*Carpinus caroliniana*), and eastern redbud (*Cercis canadensis*).

**Fig 1:**
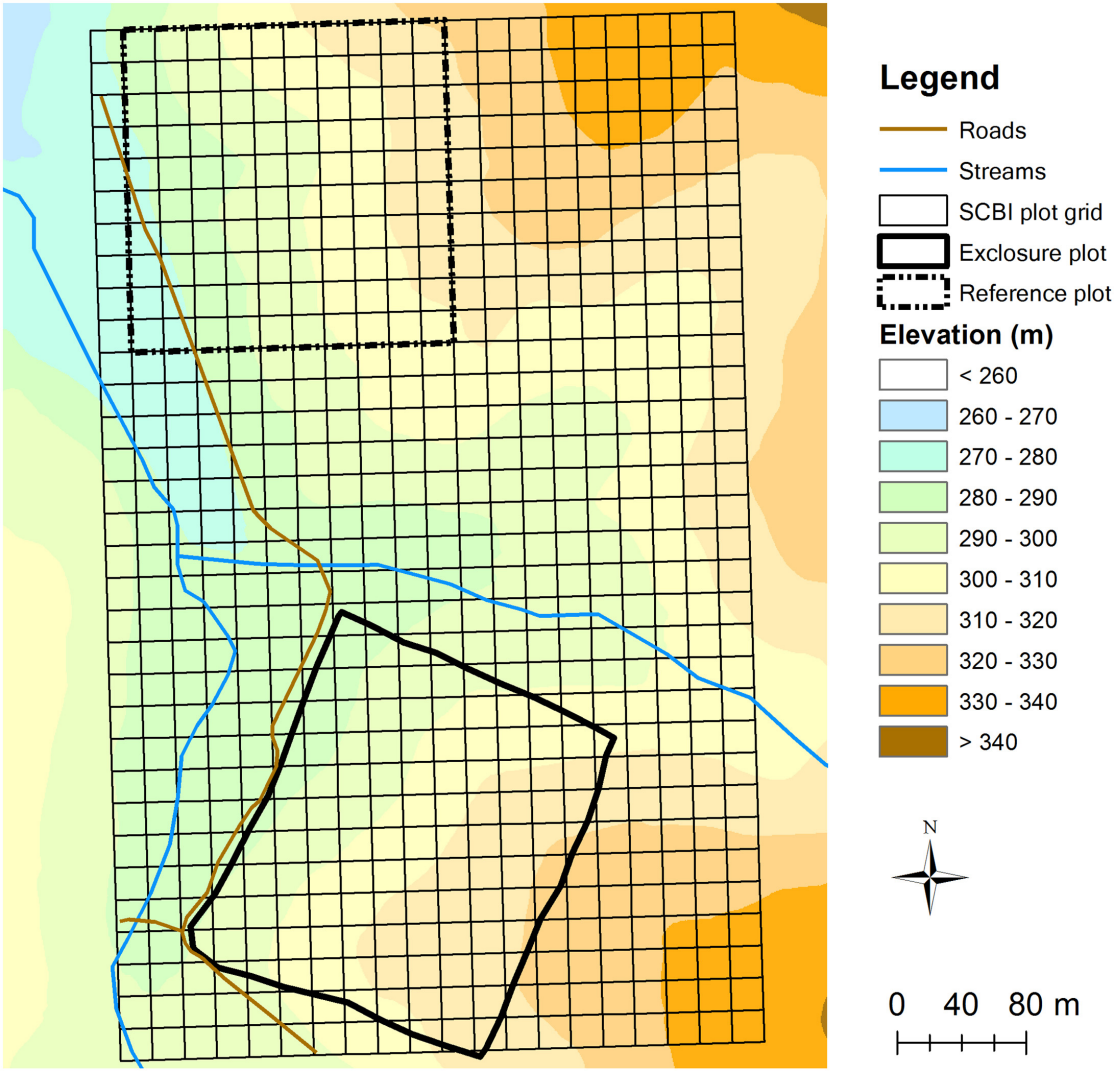
Map of the 25.6-ha (400 m x 640 m) SCBI forest plot as divided into 20 m x 20 m quadrats. The locations of the exclosure and reference plots are indicated.

The SCBI plot was gridded into 640 20 m x 20 m quadrats using iron rebar posts (Fig 1). Within the plot there is embedded a 4-ha (200 m x 200 m, containing 92 20 m x 20 m quadrats completely within the exclosure) fenced site that has excluded deer since 1991. This exclosure is constructed of woven American wire, topped with six strands of high tensile fence wire for a total height of 2.4 m, and supported by 2.4 m wooden posts at regular intervals. A 4-ha reference site (containing 100 20 m x 20 m quadrats) was identified in the SCBI plot as a comparison area for the deer exclosure. The location of the reference plot was determined by choosing an equal-sized area to the deer exclosure with similar topographic attributes (i.e., slope and aspect) and overstory composition (i.e., basal area by species), achieved by performing a cluster analysis of the ten dominant canopy tree species in each quadrat (see McGarvey et al. 2013 [8] for details).

White-tailed deer have been the only large herbivore present within the forest for at least the past century. Deer density in the SCBI forest has been consistently estimated at 30–40 deer / km^2^ over the past thirty years [26].

### Data collection

We conducted surveys to record the number of individuals of our study species within each quadrat, with the exception of the exotic invasive grass *M*. *vimineum*, where we visually estimated % cover in the quadrat. All four exotic invasive species colonized this forest after the construction of the deer exclosure in 1991 and were not detected in initial plant surveys of the area [9]. Surveys were conducted during the summer months for three species (i.e., *R*. *multiflora*, *B*. *thunbergii*, and *R*. *phoenicolasius*) in 2009, one species (*M*. *vimineum*) in 2010 and one species (*C*. *virginianum*) in 2011. During each survey, 3–4 surveyors systematically searched each quadrat across the entire SCBI plot. For *B*. *thunbergii*, *R*. *multiflora* and *R*. *phoenicolasius*, which can be multi-stemmed shrubs, we considered all stems emanating from a distinct rooting point as an individual. For *M*. *vimineum*, we estimated its % cover in five categories: 0 = absent, 1 = 0–25%, 2 = 26–50%, 3 = 51–75%, 4 = 76–100%.

For each quadrat we collected or derived 10 variables for further analysis as predictors that could potentially determine the abundance and distribution of the study species (Table 1). We estimated distance from quadrat center to forest edge, as well as the aspect, slope and landform using a 10-m Digital Elevation Model (DEM, MassGIS website). We standardized the aspect of each quadrat, calculated as “-1 x cos (45—aspect)” [28] (range -1 to 1). For landform we used a Topographic Convergence Index (TCI) [29], which is calculated as:

TCI = ln(α/tanβ)

where α is the upslope contributing area (the number of grid cells that might contribute water drainage to the point) and β is the local slope angle. TCI is positively related to water availability owing to drainage from upslope areas. Topography Toolbox 9.3 [30] embedded in ArcGIS 9.3 (ESRI, Redlands, CA, USA) was used to compute the TCI of each 10-m pixel and average TCI within each quadrat.

**Table 1:** The abundance measures (as per quadrat) of the five study species and the variables included in the generalized linear models.

	Min	Max	Mean	SD
*SPECIES ABUNDANCE*				
*Woody (No*. *of individuals)*				
Exotic invasive				
*Rosa multiflora*	0	33	1.01	2.96
*Berberis thunbergii*	0	138	6.44	11.79
*Rubus phoenicolasius*	0	125	6.33	12.96
*Herbaceous*				
Native				
*Cynoglossum virginianum (No*. *of individuals)*	0	116	10.99	18.73
Exotic invasive				
*Microstegium vimineum (categorical %) cover)*	0	4	1.76	1.2
*PREDICTOR VARIABLES*				
EDGE				
Distance to forest edge (m)	21	523	282.89	115.5
ASPECT				
Transformed aspect	-1	1	-0.009	0.72
SLOPE				
Slope (°)	1.9	21.4	10.34	3.81
TCI				
Topographic Convergence Index	0	280.8	34.45	30.35
WRICH				
Woody species richness	3	19	10.07	2.81
WSTEM				
No. of woody stems	7	582	60.37	71.22
CANOPY				
Canopy openness (%)	3.69	20.8	9.07	2.16
pH				
pH	3.96	6.11	5.09	0.47
N				
Nitrogen (NH_4_^-^ and NO_3_^-^, mg/kg)	2.13	9.09	4.30	1.17
P				
Phosphorus (mg/kg)	10.31	35.05	20.10	5.82

SCBI plot tree census data were used to calculate woody species richness and number of woody stems (≥ 1 cm DBH) as plant community attributes of each quadrat. Biophysical factors measured included canopy openness, soil characteristics, and nutrients (i.e., pH, nitrogen, and phosphorus).

An index of canopy openness in each quadrat was derived from hemispherical photographs [31] taken at 1.5 m above the ground using a Nikon FC-E8 Fish-Eye lens converter mounted on a Nikon E995 camera body. We took one horizontally leveled photograph at the center of each quadrat, either in early morning or late afternoon to avoid direct solar radiation. Photographs were interpreted using the image analysis software Gap Light Analyzer (GLA, version 2.0) [31] to obtain the percentage of open sky for each quadrat. Posthoc examination resulted in excluding those photographs taken under direct solar radiation and we obtained values for 412 quadrats.

Soil characteristics and nutrient values concentrations in the mineral topsoil (at 0–10 cm depth) were obtained at 300 points systematically located in the SCBI plot in 2010. Two hundred of the sampled locations were arrayed throughout the plot by using a regular grid of points at 33 m intervals. Each alternate grid point was paired with an additional sampling point at 2, 8, or 20 m away at a random compass bearing from the grid point to capture the variation in soil properties at finer scales. At each sample point, we collected the topsoil from approximately five cores (2.5 cm diameter). Nitrogen was extracted from fresh soil immediately following collection in 2.0 M KCI and determined as NH_4_^-^ and NO_3_^-^ by automated colorimetry on a Lachat Quikchem 8500 (Hach Ltd, Loveland, CO). Moisture was calculated by drying at 105°C. Soil pH was determined in a 1:2 soil-to-deionized water ratio using a glass electrode. Readily-extractable P, a measure of plant-available phosphate, was extracted from air-dried soil in Bray-1 solution, with detection by automated molybdate colorimetry. All soil variables were extrapolated for each 10 x 10 m grids in the plot by kriging the known values [32], and we used the averaged value of 4 10 x 10 m grids within each quadrat.

### Data analysis

We fitted generalized linear models (GLMs) to determine the most informative factors explaining the variation in abundance of our study species across the study plot. Poisson regression models were applied for *Microstegium vimineum* and negative binomial regression models were used for the other four species because their abundances exhibited over-dispersion. We used a two-step procedure when examining the effect of deer on the distribution of the study species. First, we fitted a GLM for each species with the data from the larger plot quadrats (n = 426) excluding quadrats found inside the exclosure (n = 92) and reference (n = 100) plots. After identifying significant predictor variables for the larger plot, we then constructed a second GLM for each species using the values of the relevant significant variables from the exclosure and reference plots and the additional covariate of presence/absence of deer (referred to as “FENCED” in our models). The reason we used this two-step procedure was to take advantage of the large dataset of the forest plot and identify the significant variables affecting the abundance of the species using a suite of quadrats over the broadest area possible. As the sample sizes within the exclosure (FENCED) and reference plots were relatively small (n = 192), the first step narrowed the suite of potential variables and ensured only those species-specific variables be entered into the second models. For each species-specific model we verified that the range of values for all predictor variables used in the second GLM were within the range encountered during the first phase of modeling. Twenty-two quadrats that were split by the exclosure fence (i.e., only partially inside the exclosure) were not included in either of the two models.

As the quadrats were arrayed within a grid, we investigated spatial autocorrelation of species abundance by calculating Moran’s *I* [33] using the ape package within the R statistical analysis program (R Development Team, 2008). The abundance of all species in the quadrats were spatially auto-correlated (*p* < 0.05) both within the 426 unfenced quadrats and the 192 fenced and reference quadrats, respectively, except for the abundance of *B*. *thunbergii* within the fenced and reference quadrats (*p* = 0.63 for fenced quadrats and 0.66 for reference quadrats). Thus, we added an autocovariate term (AUTOCOV_i_), a distance-weighted function of neighboring response variables [34] in all the GLMs to account for spatial correlation among neighboring quadrats, except for the second model of *B*. *thunbergii*. AUTOCOV_i_ is calculated as:

$$AUTOCOV_{i}\;=\frac{\sum_{j=1}\frac{y_{i}}{d_{ij}}}{\sum_{j=1}\frac{1}{d_{ij}}}$$

where y_j_ is the response value of y at quadrat j among quadrat i’s neighbors, in our case, eight surrounding quadrats were the neighbors of the centered quadrat; d_ij_ is the distance between the center of quadrat j and quadrat i.

We did not take interspecific relationships of the invasive species into account in the modeling process as Spearman’s correlation tests showed that the abundances of invasive species were not highly correlated with each other among quadrats (*p* > 0.05, not correlated among fenced quadrats; *r* < 0.32 among unfenced quadrats. S1 Table).

All the GLMs were constructed in the R software, and negative binomial regression models were constructed utilizing the MASS package. During the first phase of model selection, we examined the performance of each site covariate individually and retained the 6–8 variables whose model had the lowest Akaike Information Criterion value (AIC; [35]) in order to restrict the set of candidate models. We then ran models with all possible combinations of the retained site covariates and selected the best models according to AIC value. All models whose ΔAIC ≤ 2 were considered as equivalent best models [36]. A final GLM with selected variables with most occurrences from the likely models was run to identify the effects of species-specific variables for both steps. We conducted Chi-squared test using residual deviance to evaluate the goodness of fit of each model. We reported the final models from the two-step procedure for each species and a significance level of 0.05 was used for all statistical tests.

## Results

Among the three exotic invasive woody species, *B*. *thunbergii* was most abundant (4,120 individuals) and was found in 491 (77%) quadrats across the SCBI plot, including 46 (50%) in fenced and 90 (90%) in reference quadrats. *R*. *phoenicolasius* (4,052 individuals) was found in 367 (57%) quadrats, with 13 (14%) in fenced and 78 (78%) in reference quadrats. *R*. *multiflora* (646 individuals) was found in 188 (29%) quadrats, including 27 (29%) in fenced and 18 (18%) in reference quadrats. The exotic invasive herbaceous *M*. *vimineum* was widespread in 551 (85%) quadrats (44 (48%) in fenced and 98 (98%) in reference quadrats) at an average categorical cover value of 1.8, while the native *C*. *virginianum* was recorded as 1,642 individuals in 299 (47%) quadrats (1 (1%) in fenced and 85 (85%) in reference quadrats) (Table 1).

### Species-specific relationships with deer abundance

The abundance of the native species *C*. *virginianum* across the larger plots was best predicted by EDGE, SLOPE, TCI, pH and AUTOCOV (Table 2). More *C*. *virginianum* individuals were found in quadrats further away from the forest edge, in drier places, and on the slopes than in flatter areas. Higher soil pH was correlated with decreased abundance of *C*. *virginianum*. When we examined the matched quadrats inside and outside the deer exclosure, EDGE, SLOPE and AUTOCOV were retained in the second negative binomial regression model, and deer presence/absence (FENCED) was an influential factor determining its abundance. *C*. *virginianum* plants were found 250 times more often in the reference quadrats than in the deer-free quadrats.

**Table 2:** Parameter estimates, standard errors and *p*-values (*** <0.001, ** <0.01, *<0.05,. <0.1) for final generalized linear models estimating the effects of site covariates on the abundance of native *Cynoglossum virginianum* in the SCBI forest plot.

	*Cynoglossum virginianum* (CYVI)			
	Estimate(*β*)	SE	*p*	Exp.(*β*)
*Step 1*: *within unfenced*, *non-reference area*				
EDGE	0.006	0.0008	***	1.006
SLOPE	0.064	0.022	**	1.066
WRICH				
WSTEM				
TCI	-0.009	0.003	**	0.991
pH	-0.392	0.169	*	0.676
N				
CANOPY				
Autocov	0.059	0.005	***	1.061
Model fit: res.deviance = 357.1588, df = 340, *p* = 0.25				
*Step 2*: *within deer-free (fenced) and reference areas*				
EDGE	0.012	0.003	***	1.012
SLOPE	0.128	0.044	**	1.137
N				
Autocov	0.049	0.011	***	1.05
FENCED	-5.519	1.023	***	0.004
Model fit: res.deviance = 126.286, df = 185, *p* = 0.99				

The most predictive factors for *R*. *multiflora* in the larger plot were EDGE, SLOPE, and AUTOCOV (Table 3A). Soil pH and N were in the final negative binomial regression model but their effects were not significant. The quadrats with higher *R*. *multiflora* abundance were found near the forest edge and in flatter areas. For the comparison of exclosure and reference quadrats, the abundance of *R*. *multiflora* was predicted by presence/absence of deer (FENCED) and N. The number of *R*. *multiflora* in the deer-free quadrats was 3.8 times that of the reference quadrats.

**Table 3:** Parameter estimates, standard errors and *p*-values (*** <0.001, ** <0.01, *<0.05,. <0.1) for final generalized linear models estimating the effects of site covariates on the abundance of four exotic invasive species in the SCBI forest plot.

**A.**								
	*Rosa multiflora* (ROMU)				*Berberis thunbergii* (BETH)			
	**Estimate(*β*)**	**SE**	***p***	**Exp.(*β*)**	**Estimate(*β*)**	**SE**	***p***	**Exp.(*β*)**
*Step 1*: *within unfenced*, *non-reference area*								
EDGE	-0.003	0.0009	***	0.997	-0.001	0.0005	*	0.999
SLOPE	-0.09	0.026	***	0.914	-0.033	0.013	*	0.968
WRICH					0.029	0.02		1.029
WSTEM								
TCI								
pH	0.432	0.224	.	1.54	0.385	0.114	***	1.47
N	0.152	0.103		1.164				
CANOPY	0.077	0.022	***	1.08	0.021	0.011	.	1.021
Autocov	0.167	0.043	***	1.182	0.073	0.006	***	1.076
Model fit: ROMU: res.deviance = 311.9267, df = 419, p = 0.99; BETH: res.deviance = 484.699, df = 419, p = 0.02								
*Step 2*: *within deer-free (fenced) and reference areas*								
EDGE								
SLOPE								
N	0.359	0.138	**	1.432				
Autocov								
FENCED	1.343	0.373	***	3.831	-0.592	0.164	***	0.553
Model fit: ROMU: res.deviance = 117.6605, df = 189, p = 0.99; BETH: res.deviance = 214.9352, df = 190, p = 0.10								
**B.**								
	*Rubus phoenicolasius* (RUPH)				*Microstegium vimineum* (MIVI)			
	**Estimate(*β*)**	**SE**	***p***	**Exp.(*β*)**	**Estimate(*β*)**	**SE**	***p***	**Exp.(*β*)**
*Step 1*: *within unfenced*, *non-reference area*								
EDGE								
SLOPE					-0.022	0.009	*	0.978
WRICH								
WSTEM					-0.001	0.0006	.	0.999
TCI								
pH	0.453	0.161	**	4.348				
N	0.253	0.078	**	1				
CANOPY	-0.033	0.016	*	2.777				
Autocov	0.085	0.007	***	2.932	0.474	0.042	***	1.606
Model fit: RUPH: res.deviance = 449.5587, df = 421, p = 0.16; MIVI: res.deviance = 194.6704, df = 422, p = 1								
*Step 2*: *within deer-free (fenced) and reference areas*								
EDGE								
SLOPE								
N	0.28	0.091	**	1.323				
Autocov	0.139	0.035	***	1.149	0.465	0.124	***	1.592
FENCED	-1.565	0.283	***	0.209	-0.675	0.257	**	0.509
Model fit: RUPH: res.deviance = 170.5647, df = 188, p = 0.81; MIVI: res.deviance = 100.5712, df = 189, p = 1								

The significant variables retained in the *B*. *thunbergii* model for the larger plot were EDGE, SLOPE, pH and AUTOCOV (Table 3A), among which EDGE and SLOPE had negative effects and pH had positive effects on the abundance of *B*. *thunbergii*. For the comparison of exclosure and reference quadrats, only deer presence/absence (FENCED) was a significant predictor. The number of *B*. *thunbergii* within the reference was1.8 times that of the deer-free quadrats.

The predictive factors in the *R*. *phoenicolasius* model for the larger plot were pH, N, CANOPY and AUOTCOV (Table 3B). Soil pH and N were positively associated with the abundance of *R*. *phoenicolasius*, while a negative relationship was found between canopy openness and *R*. *phoenicolasius* abundance. For the comparison of exclosure and reference quadrats, N and AUTOCOV were retained in the model, and deer presence/absence (FENCED) was identified as important. The number of *R*. *phoenicolasius* in the reference plot was 4.8 times that of the deer-free plot.

The significant variables retained in the *M*. *vimineum* model for the larger plot were SLOPE and AUOTCOV (Table 3B). There were higher levels of % coverage for quadrats in flatter areas. The comparison of exclosure and reference quadrats resulted in a final model with just deer exclosure (FENCED) as explanatory. The average categorical cover of *M*. *vimineum* in the reference was about 2 times that of the deer-free quadrats.

## Discussion

### Important predictors of species abundance

Our species-specific models for the entire 25.6- ha plot identified landscape and soil parameters related to the abundance of the species. Among the variables examined, slope, distance to forest edge and soil pH was the most frequent predictors of the abundance of the focal species on the larger plot. The deviance goodness-of-fit test indicated that all the GLMs fitted the data well, except for the first step model of *B*. *thunbergii* where the environmental and landscape variables had weak effects on its abundance. The autocovariate term, AUTOCOV, was retained in most models and was a highly significant predictor of the abundance of the study species, indicating a strong spatial correlation of their abundance among neighboring quadrats.

Slope was an important predictor of the abundance of four of our study species (*C*. *virginianum*, *R*. *multiflora*, *B*. *thunbergii* and *M*. *vimineum*). Indeed, it was identified as the only important predictor of *M*. *vimineum* abundance. SLOPE was correlated with TCI (*r* = -0.564, *p* < 0.001) and its effects on the abundance of the four species may reflect the influence of soil water level on the abundance of these species. TCI was not related to the abundance of any of our exotic invasive species (Table 3A and 3B), but correlation tests between TCI and their abundance did indicate a relationship for all but *R*. *phoenicolasius* (*R*. *multiflora*, *r* = 0.271, *p* < 0.001; *B*. *thunbergii*, *r* = 0.333, *p* < 0.001; *M*. *vimineum*, *r* = 0.214, *p* < 0.001; *C*. *virginianum*, *r* = -0.341, *p* < 0.001). *C*. *virginianum* is an upland species preferring dry soils, confirmed by the negative relationship between TCI and *C*. *virginianum* abundance (Table 2, *β* = -0.009, *p* < 0.05). The other three exotic invasive species (*R*. *multiflora*, *B*. *thunbergii* and *M*. *vimineum*) all preferred flatter areas, which in our plot were the riparian areas, a nutrient-rich habitat, and swales at the bottom of slopes. The higher abundance of these species in these areas was possibly attributable to the higher seed or fruit densities carried by water, which is especially true for *M*. *vimineum* [37], or a more optimal surface for their seed germination provided by richer soils and more moisture [38,39].

Distance to the forest edge was another important predictor of the abundances of three of our study species (*C*. *virginianum*, *R*. *multiflora* and *B*. *thunbergii*). Similar to slope, the direction of this predictor varied between the native *C*. *virginianum* (positively associated with its abundance) and the two exotic invasive species (negatively associated). Beyond being the possible entry point of invasion for exotic species, the edge is associated with increased anthropogenic disturbance and resource availability [40]. In our study site, soil nitrogen and water (as measured by TCI) levels decreased with distance into the forest (Spearman correlation, *r* = -0.339, *p* < 0.001; *r* = -0.158, *p* < 0.001 respectively). The presence of a stream along the western portion of the SCBI plot resulted in the quadrats in this area having both proximity to the forest edge and high soil moisture. Increased nitrogen, water and light along the forest edge would foster the invasion of *R*. *multiflora* and *B*. *thunbergii* and a positive edge effect has been described for both species [41,42]. For *C*. *virginianum*, a long-lived and shade-tolerant summer perennial [25], drier soils in the forest interior may lead to its higher abundance.

Soil pH was another frequent model variable and important predictor of the abundance of three of our study species (*C*. *virginianum*, *B*. *thunbergii*, and *R*. *phoenicolasius*). At our forest (pH = 3.96–6.11), lower pH soils were preferred by the native *C*. *virginianum*, while higher pH soils were preferred by the two exotic invasive species (*B*. *thunbergii* and *R*. *phoenicolasius*). Ehrenfeld et al (2001) demonstrated that the invasion of *B*. *thunbergii* can elevate soil pH via nitrate uptake, and such changes may establish a positive feedback system that further enhances its invasion [11]. While we could find no studies relating pH to *R*. *phoenicolasius* abundance, it is well known in agricultural production that *Rubus* species prefer a soil pH range of 5.6–6.5 [43,44].

Other variables that were correlated with the abundance of our study species were extractable N and canopy openness. Nitrogen was the important predictor of *R*. *phoenicolasius* abundance, and as previous studies show, high N concentrations facilitated its invasion [45]. Higher canopy openness led to higher abundance of *R*. *multiflora*, but unexpectedly, canopy openness was negatively associated with the abundance of *R*. *phoenicolasius*. Gorchov et al. (2011) found that *R*. *phoenicolasius* required large canopy gaps to establish in mature Mid-Atlantic deciduous forest, but established plants could survive canopy closure at light levels as low as 5% full sun [46]. Given that canopy openness of our forest was low across the plot (9.0% ± 2.1%), large canopy gaps required for establishment were largely lacking and *R*. *phoenicolasius* was more dispersed in the understory. Although light has long been recognized as an important plant resource in the temperate forest understory [47], it was not correlated with most of our study species.

### Deer as an important driver of exotic plant invasion

When we included the presence of deer as a covariate in our models for each species, regardless of the environmental variables added into the models, FENCED was a highly significant predictor of the abundance of all species and its effect was the strongest (stronger than the AUTOCOV term) for most of the species. The native plant *C*. *virginianum*, and the exotic plants *B*. *thunbergii*, *R*. *phoenicolasius* and *M*. *vimineum*, were more abundant where deer were present, whereas *R*. *multiflora* was less abundant in the presence of deer.

We included the native *C*. *virginianum* in this study because deer are seed-dispersal agents for this species [25]. Although most seeds of *C*. *virginianum* are found near the parental plant [25], occasional seed dispersal events by animals, such as white-tailed deer, are important for its long-distance dispersal [48]. We only detected one individual of *C*. *virginianum* in the exclosure and it was located directly next to the fence. *C*. *virginianum* is in the Boraginaceae, a family with pyrrolizidine alkaloids [49], which can protect plants to some degree from generalist herbivores [48] and *C*. *virginianum* is not a preferred forage plant of deer [50]. *C*. *virginianum* was present within the exclosure prior to fence erection [9]; its loss from the area indicates that, in addition to limited dispersal, the loss of deer removed any competitive advantage derived from its chemical defenses and deer-assisted dispersal.

White-tailed deer significantly affected the abundance of the four exotic invasive species. One exotic invasive species, *R*. *multiflora*, had a higher density within the exclosure than in the reference quadrats, indicating little or no role for deer in facilitating invasion. The distribution pattern of *R*. *multiflora* within the exclosure indicated its occurrence was stochastic. The fence was constructed prior to the colonization of this exotic species and there was no significant correlation between plant density and distance to the fence (*r* = -0.103, *p* = 0.329). *R*. *multiflora* is mainly dispersed by birds and Stiles (1982) [51] presents a strong association between the range expansions of *R*. *multiflora* and the northern mockingbird (*Mimus polyglottos*) in North America. Seeds of *R*. *multiflora* are also preferred food items for white-footed mice (*Peromyscus leucopus*) [52], a common species in our forest [53]. Studies at the same site found higher abundances of small mammals [53] and birds [9] in the exclosure relative to the reference site as understory vegetation advanced through successional processes after deer exclusion. Thus, it is possible that higher density of *R*. *multiflora* in the exclosure is attributable to the higher activities of its primary dispersal agents (i.e. birds and small mammals), which facilitated its dispersal and colonization by ingestion and defecation or caching in the exclosure.

For three exotic invasive species, the absence of deer was negatively correlated with their abundance, and for *B*. *thunbergii* and *R*. *phoenicolasius*, FENCED was the only predictor of their abundance within exclosure and reference quadrats. These results suggest that deer contributed more to predicting the abundance of exotic invasive plants in our forest than did abiotic factors (i.e., nitrogen, light, water and distance to the forest edge) at the local scale. The AUTOCOV term as a predictor of the abundance of these three species in the species-specific models indicated that they spread from the established sites, and the fence lines did slowdown their rate of spread into the deer exclosure. Our results concur with other studies [13,18] showing that deer acted as facilitators for the invasion of *B*. *thunbergii*, *R*. *phoenicolasius* and *M*. *vimineum*. They also agree with our subsequent observations in summer 2011, of the occurrence of wavyleaf basketgrass (*Oplismenus hirtellus* ssp. *undulatifolius*), a new exotic invasive species [54], in the reference plot and its absence within the exclosure (N.A. Bourg, unpublished data). If deer facilitated the expansion of three exotic invasive species, we hypothesize four potential causal mechanisms including endozoochory, changes to soil nutrients, selective browsing, and increased resistance to invasion of local communities.

Endozoochory by deer might facilitate the dispersal and establishment of the exotic invasive species in our forest as recent studies found viable seeds of *R*. *multiflora*, *R*. *phoenicolasius* and *M*. *vimineum* in deer fecal pellets [16,17]. Myers et al. (2004) identified 72 taxa germinated from deer feces collected in mixed deciduous forests in Ithaca, New York, including *R*. *multiflora* and *Rubus* spp. (frequency in pellet groups: 2% and 3% respectively) [16]. Williams et al. also recorded *R*. *multiflora*, *R*. *phoenicolasius* and *M*. *vimineum* (2%, 6% and 0.5% respectively) as among the 86 taxa germinated from deer pellets collected on a forested water authority property in southern Connecticut [17]. Deer also occasionally consume the fruits of *B*. *thunbergii* [55]. Deer may not be the major dispersal agent of our study species (e.g., *R*. *multiflora* and *B*. *thunbergii* mainly by birds and *M*. *vimineum* by surface water runoff), but considering the dense populations of white-tailed deer in our forest, the importance of deer ingestion in dispersing these species into forest interiors should be considered.

A comparison of soil nutrient levels between the exclosure and reference quadrats indicated higher nitrogen and phosphorus levels in the reference quadrats (Mann-Whitney U test, Z = -3.022, *p* = 0.003; Z = -11.153, *p* < 0.001 respectively). Several studies have found that white-tailed deer and other cervids (e.g., *Capreolus capreolus*) transfer nutrients from adjacent land types into forest patches [56, 57]. Deer may be the source of higher nitrogen and phosphorus levels in the reference quadrats, thereby increasing susceptibility of the native community in the reference area to invasion.

An indirect route by which deer could have facilitated invasion by the three exotic plants is via selective browsing. Sustained browsing pressure in forests of North America has shifted herbaceous and woody plant composition towards unpalatable and/or browse-resistant species (including invasive species) and led to low species abundance and diversity [58]. Selective herbivory of preferred native species over exotic species, compounded with other disturbances by deer (e.g., trampling [19]; soil compaction [59]), would release exotic invasive plants from intense competition with native species by increasing availability of resources such as open space, light, and soil nutrients. A study of woody species at our site [8] suggested 20-year deer browsing has remarkably suppressed seedling height growth and small sapling abundance in the reference quadrats with seedling height on average 2.25-times greater and sapling (1–5 cm DBH) stem counts 4.1-times greater inside the exclosure. Heckel et al. also documented larger plant sizes and higher population viability of five unpalatable herbaceous species (e.g., *Arisaema triphyllum*, *Actaea racemosa* and *Osmorhiza claytonii*) in the exclosure relative to paired deer access plots at our study site [59].

The deer exclosure was constructed prior to the colonization of the exotic species in our forest. As discussed above, the exclusion of deer since 1991 has increased the density and diversity of understory woody plants relative to the reference quadrats [8,9], and possibly increased the abundance and diversity of herbaceous species as well [59]. Higher native species diversity does confer greater community-level resistance to invasion, because species-rich communities more completely and efficiently use available resources [60, 61]. Kalisz et al. observed that excluding deer increased the growth rate of a native forb, *Trillium erectum*, leading to the restoration of the potent biotic resistance of the native community to the exotic invasive *Alliaria petiolata* [14]. The exclusion of deer prior to invasive arrival may have made the exclosure site more resistant to exotic plant invasion through the increased growth of the native plant community.

Although it is generally assumed that intact forests are highly resistant to plant invasions [24], our long-term large-scale deer exclusion experiment shows that exotic invasive plants were able to penetrate into the forest with the assistance of anthropogenic disturbance, even when the deer were excluded from the forest prior to their invasion. All exotic invasive species had established in the exclosure at the time of our survey. *R*. *multiflora*, *B*. *thunbergii*, and *R*. *phoenicolasius* were present in 29%, 50% and 14% of the fenced quadrats, respectively, in 2009 and *M*. *vimineum* was present in 48% of fenced quadrats in 2010. A resurvey of these species in the exclosure in 2014 found fast spread of *R*. *multiflora* and *B*. *thunbergii* (present in 63% and 89% fenced quadrats, respectively). The distribution of *R*. *phoenicolasius* remained stable (12%) and that of *M*. *vimineum* shrank (17%) as the drought in subsequent year reduced its coverage. The absence of these exotic invasive plants in our forest before the establishment of the deer exclosure and their colonization and fast spread in the exclosure 20 years later suggest that even when deer densities can be maintained at a relatively low level, it alone will not be enough to prevent exotic invasive plants from spreading into forest communities.

## Conclusions

Our study highlighted the important role of white-tailed deer in determining the distribution and abundance of several exotic invasive species. Although multiple factors, such as slope, edge effects, and soil pH, influenced the distribution and abundance of our study species, deer presence contributed more strongly to predicting their abundance at a local scale. It reflected the cumulative effects of sustained deer browsing and movement on the distribution of the study plants in our forest over the past 20 years. These interactions between deer and exotic species may accelerate the invasion of exotic species into similar forests in the Mid-Atlantic region of North America.

## Supporting Information

S1 Table Spearman’s rank correlation coefficient of the abundance of invasive species in each quadrat within the SCBI forest plot. (DOCX)

S2 Table Abundance of the study species and environmental variables used in the generalized linear models. (XLSX)
