# Supplementary material for: Long-Term Effects of White-Tailed Deer Exclusion on the Invasion of Exotic Plants: A Case Study in a Mid-Atlantic Temperate Forest
Source: PLoS One. 2016 Mar 28;11(3):e0151825. doi: 10.1371/journal.pone.0151825 (PMC4809546; doi:10.1371/journal.pone.0151825)
Supplement: S1 Table — (DOCX) [file pone.0151825.s001.docx]

Appendix S1. Correlations of the abundance of invasive species in each quadrat within the SCBI forest plot

1. Among fenced quadrats (n=92)

|  | ROMU | BETH | RUPH | MIVI |
| --- | --- | --- | --- | --- |
| ROMU | 1 | 0.114 | 0.098 | -0.089 |
| BETH | 0.114 | 1 | 0.068 | -0.034 |
| RUPH | 0.098 | 0.068 | 1 | -0.106 |
| MIVI | -0.089 | -0.034 | -0.106 | 1 |

1. Among unfenced quadrats (n=526)

|  | ROMU | BETH | RUPH | MIVI |
| --- | --- | --- | --- | --- |
| ROMU | 1 | .314^**^ | .172^**^ | .128^**^ |
| BETH | .314^**^ | 1 | .120^**^ | .147^**^ |
| RUPH | .172^**^ | .120^**^ | 1 | .268^**^ |
| MIVI | .128^**^ | .147^**^ | .268^**^ | 1 |
